# Post–Endoscopic Retrograde Cholangiopancreatography (ERCP) Subcapsular Hepatic Hematoma

**DOI:** 10.7759/cureus.31691

**Published:** 2022-11-20

**Authors:** Ayham Khrais, Ahmad Ali, Catherine Choi, Siddharth Verma

**Affiliations:** 1 Department of Medicine, Rutgers New Jersey Medical School, Rutgers University, Newark, USA; 2 Department of Neurology, Rutgers New Jersey Medical School, Rutgers University, Newark, USA; 3 Department of Gastroenterology and Hepatology, Rutgers New Jersey Medical School, Rutgers University, Newark, USA; 4 Department of Gastroenterology and Hepatology, East Orange Veterans Affairs Medical Center, East Orange, USA

**Keywords:** bile duct diseases, liver hematoma, adult gastroenterology, upper endoscopy, endoscopy ercp

## Abstract

Physicians need to recognize the potential complications of endoscopic retrograde cholangiopancreatography (ERCP), which are rare but can be serious. We describe a case of post-ERCP subcapsular hepatic hematoma (SHH). A 39-year-old man with a history of alcohol use, complicated by chronic pancreatitis and common bile duct (CBD) stricture, presented with right upper quadrant pain two weeks after the placement of a stent for CBD stricture. He was managed with pain control and antibiotics. SHH is a rare complication of ERCP. Hematomas can expand, resulting in significant anemia and liver function test (LFT) elevation, or can become infected. Patients with SHH must be carefully monitored in the post-ERCP setting.

## Introduction

Endoscopic retrograde cholangiopancreatography (ERCP) is a diagnostic and therapeutic procedure indicated for managing biliary and pancreatic pathology, including choledocholithiasis, biliary strictures, and gallstone pancreatitis [1,2]. Despite its clinical benefits and utility, ERCP has a significant complication profile, including post-ERCP pancreatitis, cholangitis, cholecystitis, perforation of a hollow viscus, and bleeding [3,4]. Bleeding complications range from mild traumatic mucosal and submucosal hemorrhage to symptomatic blood loss from organ injury requiring multiple transfusions. Patients on antiplatelet agents, anticoagulants, and with coagulopathy are at higher risk of bleeding; however, with certain procedural techniques, mid-ERCP can also increase the risk of bleeding, including uncontrolled *zipper* cuts, sphincterotomy, and use of a needle-knife sphincterotome [5,6]. Bleeding can occur due to papillary manipulation and guidewire-induced trauma. Subcapsular hepatic hematoma (SHH) is a rare yet potentially devastating complication of ERCP, with as few as 20 described cases [7]. The hypothesized mechanism of injury leading to SHH involves perforation of the intrahepatic biliary tree walls during tool advancement (i.e., guidewires), resulting in traumatic rupture of small intrahepatic blood vessels [7,8]. Blood can pass through liver parenchyma to form hematomas in dependent portions of the organ, with blood collections being contained by the liver capsule. While many patients with ERCP-induced SHH remain hemodynamically stable, there is a risk of hematoma infection or expansion, leading to significant anemia [7,8]. Therefore, patients with SHH must be identified quickly and monitored serially to prevent potential decompensation and sepsis. Here, we describe a case of ERCP-induced SHH managed conservatively.

## Case presentation

**Figure 1:**
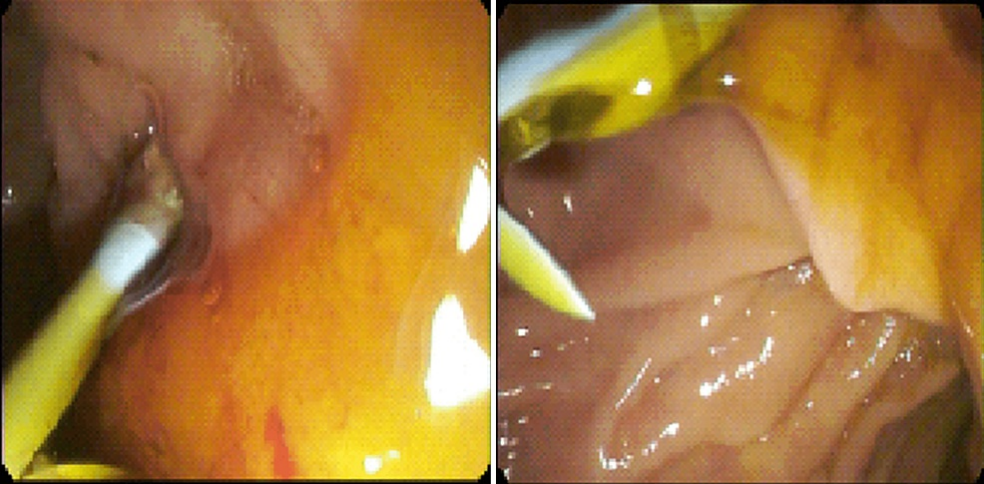
Endoscopic retrograde cholangiopancreatography images demonstrating the placement of a new 10 French × 9 cm plastic stent (left) and subsequent flow of bile (right).

A 39-year-old man with a history of hypertension, type II diabetes mellitus, post-traumatic stress disorder, tobacco use disorder, and alcohol use disorder complicated by alcoholic fatty liver disease and chronic pancreatitis with multiple episodes of CBD stricture status post multiple ERCPs presented with epigastric pain and right upper quadrant (RUQ) pain. His previous strictures were deemed likely secondary to fibrosis resulting from his chronic pancreatitis. Of note, the patient was admitted two weeks earlier for epigastric pain, was found to have CBD stricture, and underwent ERCP involving guidewire placement, subsequent balloon sweeps, and removal of an existing biliary stent and placement of one new 10 French x 9 cm plastic stent (Figure 1). During that previous hospitalization, his hemoglobin was at baseline. His pain did not resolve since and now involved the RUQ. The pain escalated to a 10/10, causing him to return for further management. The pain was constant, unrelieved with Tylenol, which he took at a maximum dose of 2 g/day, and Naproxen. He also felt occasional nausea but no vomiting.

**Figure 2:**
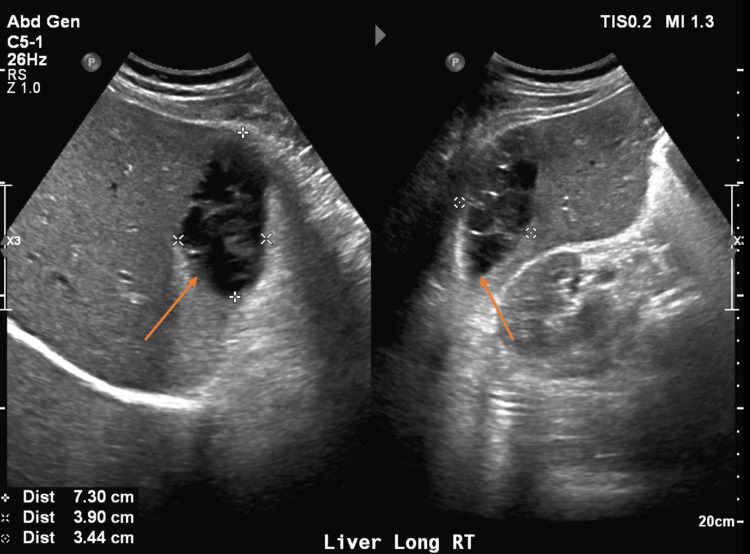
Abdominal ultrasound demonstrating an area of mixed echogenicity on the right side of the liver measuring 7.3 cm × 3.9 cm, likely a hematoma or an abscess (arrow).

**Figure 3:**
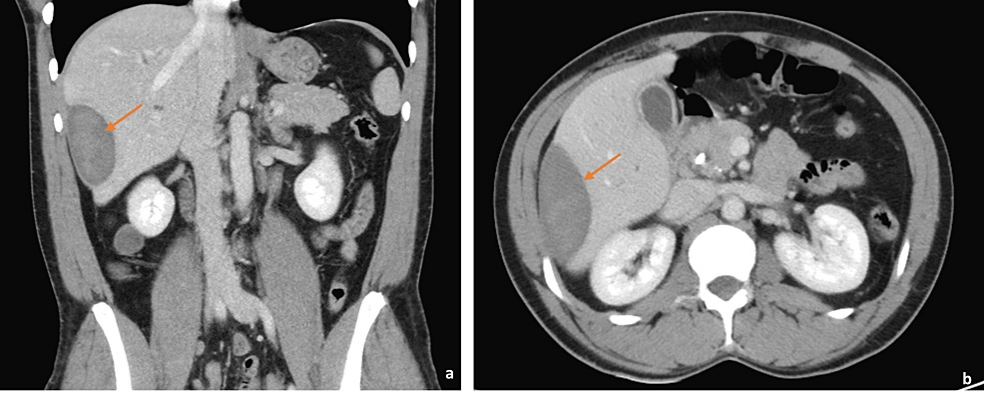
A triple-phase computed tomography scan of the abdomen and pelvis demonstrating a right-sided subcapsular hematoma in segment 6 of the liver within the (a) coronal and (b) transverse planes (arrow).

On arrival, he was afebrile and vital signs were unremarkable. Physical exam was significant for epigastric and RUQ tenderness to palpation with voluntary guarding but a soft abdomen without rebound tenderness. Laboratory findings were significant for white blood cell (WBC) count of 10.8 (reference range [RR] 4.5 to 11.0 × 10^9 ^L^-1^), alkaline phosphatase (ALP) of 117 (RR 44-147 International Units/L), hemoglobin (Hgb) 11.7 (RR 14.0 - 17.5 g/dL), which was at baseline. Bilirubin and transaminase levels were within normal limits. Abdominal ultrasound (AUS) demonstrated an area of mixed echogenicity on the right side of the liver measuring 7.3 cm × 3.9 cm with marked dilation of the common bile duct (CBD) and intrahepatic biliary tree (Figure 2), slightly decreased when compared with earlier imaging. A triple-phase CT scan of the abdomen and pelvis (A/P) was significant for a 9 cm × 6.5 cm × 2.5 cm heterogeneous, high-density collection in the subcapsular segment 6 of the liver without rim enhancement, likely representing a hematoma, as well as persistent CBD dilation of 13 mm (Figure 3). He was initially managed with conservative treatment, including Piperacillin-Tazobactam, intravenous fluids (IVFs), and pain control. His WBC count trended down. Piperacillin-Tazobactam was subsequently discontinued due to a lack of fever, leukocytosis, and other clinical signs of infection.

On hospital day 3, his liver function tests (LFTs), previously within normal limits, were elevated. Aspartate aminotransferase (AST) was 397 (RR 8-33 U/L), alanine aminotransferase (ALT) was 206 (RR 7-55 U/L), ALP was 571 (RR 44-147 U/L), and total bilirubin (TB) was 5.0 (RR 0.1-1.2 mg/dL). Patient’s urine was dark amber; however, his pain was improving. Repeat CT A/P was performed that same day to assess for change in the hepatic hematoma, demonstrating expected resolution of the hematoma without change in size, with his biliary stent in correct placement. He was restarted on Piperacillin-Tazobactam, then transitioned to Levofloxacin and Metronidazole. His pain and liver enzymes continued to improve. He remained afebrile without leukocytosis throughout his hospital course. The patient was discharged home to complete a 14-day course of antibiotics. Repeat imaging performed one week after discharge demonstrated decrease in size of the hematoma, measuring 4.9 cm × 3.4 cm × 2.7 cm.

## Discussion

SHH is a rare complication of ERCP caused by guidewire-induced trauma to intrahepatic vessels. It most commonly presents either as sole abdominal pain or as pain co-occurring with anemia or fever [7]. The pain usually occurs within 24 hours of ERCP; however, it has been shown to occur days after the procedure [9]. Imaging studies, whether ultrasound, CT, or MRI, are the definitive diagnostic modalities that can accurately assess the position and extent of the hematoma. CT scans are typically preferred as they are ubiquitous and can be performed quickly. Laboratory studies are necessary adjuncts to identify acute blood loss anemia that may have resulted from the hematoma.

Management of SHH depends on the patient’s clinical status. In the presence of hemodynamic stability, conservative treatment is preferred [10]. When identified, patients with SHH should be placed on broad-spectrum antibiotics, as hematomas can become secondarily infected, resulting in sepsis and the potential need for invasive drainage and procedures [10]. If the patient is hemodynamically unstable or septic or if the hemorrhage is actively expanding, then invasive interventions, including drainage or laparoscopy with irrigation, should be considered [10].

Here, we describe a case of SHH presenting with new-onset RUQ abdominal pain superimposed on preexisting epigastric pain. The hematoma was seen on the ultrasound and CT and was managed conservatively with antibiotics and analgesics. This case was further interesting, in that the patient’s LFTs had a delayed rise a few days after diagnosis of SHH, with subsequent spontaneous improvement. This phenomenon likely represented the resolution of the hematoma with concomitant transient injury to surrounding hepatocytes. The patient was discharged with outpatient antibiotics and follow-up imaging.

## Conclusions

SHH must be considered in patients experiencing new or different post-ERCP abdominal pain, with workup including CBC and CT scans to evaluate for possible hematoma and symptomatic anemia. As seen in our patient, treatment for SHH is mostly conservative, and patients must be serially monitored for infection or hematoma expansion.
